# Epileptic spike-wave discharges in a spatially extended thalamocortical model

**DOI:** 10.1186/1471-2202-14-S1-P87

**Published:** 2013-07-08

**Authors:** Peter N Taylor, Yujiang Wang, Gerold Baier, Sydney S Cash, Justin Dauwels

**Affiliations:** 1 School of Electrical & Electronic Engineering, Nanyang Technological University, Singapore; 2 Manchester Interdisciplinary Biocentre, University of Manchester, UK; 3 Centre for Organismal Studies, University of Heidelberg, Germany; 4 Massachusetts General Hospital and Harvard Medical School, Cambridge, USA

## 

Generalised spike-wave discharges (SWD), detectable on the electroencephalogram (EEG), are a hallmark of typical absence seizures. Several mechanisms of transmission have been proposed for SWD seizures including the centrencephalic theory, the corticoreticular theory, and the cortical focus theory [1]. Experimental evidence suggests that seizures rapidly generalise through cortico-cortical and thalamo-cortical pathways from an excitable cortical 'focus' [1]. Further evidence for a cortical focus comes from recent experimental results which show that stimulations to different cortical regions can elicit generalised SWD, however, the amplitude of the required stimulus varies from one region to another [2].

**Figure 1:**
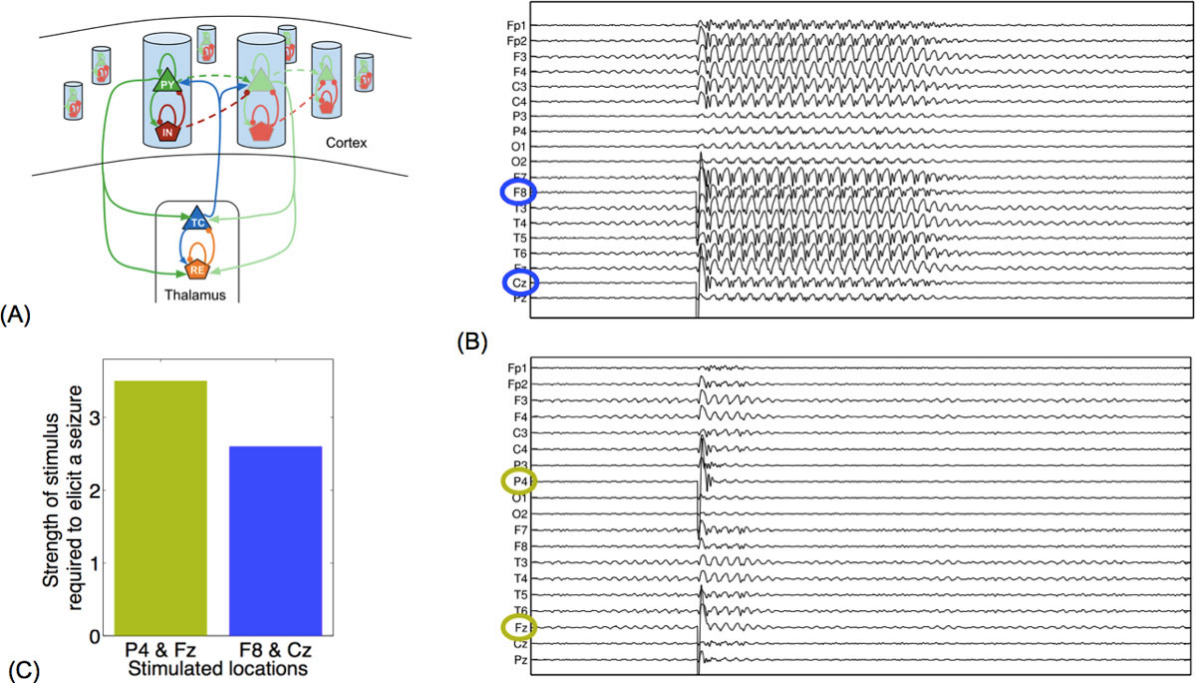
**(A) Connectivity scheme of the model**. (B) Top panel: Stimulus induced SWD seizure. Bottom panel: Stimulus induced short transient response (no seizure). (C) Minimum amplitude of stimulus required to initiate a seizure

Based on known anatomical connectivity between the thalamus and cortex (Figure. 1A), we develop a macroscopic model of transitions between inter-ictal and ictal SW dynamics to investigate the cortical focus theory. Single pulse perturbations of sufficient amplitude can drive the model into the seizure state, i.e. the dynamics are excitable. Mechanistically, the stimuli transiently drive the cortical subsystem beyond a saddle-node bifurcation (c.f. [3]).

We extend the model to include multiple cortical compartments (using cortico-cortical connectivity inferred from patient EEG) and find that the threshold for an excitable response varies between cortical regions. Figure 1B (top panel) shows an exemplary time series where a stimulus is applied which results in a long seizure-like transient. In Figure 1B (bottom panel), the stimulation of other cortical compartments elicits only short responses. In agreement with [2] a stimulus of higher amplitude is required to elicit a seizure (Figure 1C). This shows that whilst thalamo-cortical connectivity is essential for SWD maintenance, cortico-cortical connectivity crucially influences the site of SWD initiation. The model provides evidence for the cortical focus theory, where specific cortical regions are more susceptible to producing generalised SWD seizure upon stimulation. This is strongly dependent on the patient specific heterogeneous cortico-cortical and thalamocortical connectivity.
